# Receptor-transporting protein (RTP) family members play divergent roles in the functional expression of odorant receptors

**DOI:** 10.1371/journal.pone.0179067

**Published:** 2017-06-06

**Authors:** Teng Yu, Xubo Su, Yi Pan, Hanyi Zhuang

**Affiliations:** 1 Department of Pathophysiology, Key Laboratory of Cell Differentiation and Apoptosis of the Chinese Ministry of Education, Shanghai Jiao Tong University School of Medicine, Shanghai, China; 2 Institute of Health Sciences, Shanghai Jiao Tong University School of Medicine/Shanghai Institutes for Biological Sciences of Chinese Academy of Sciences, Shanghai, China; Monell Chemical Senses Center, UNITED STATES

## Abstract

Receptor transporting protein (RTP) family members, RTP1S and RTP2, are accessory proteins to mammalian odorant receptors (ORs). They are expressed in the olfactory sensory neurons and facilitate OR trafficking to the cell-surface membrane and ligand-induced responses in heterologous cells. We previously identified different domains in RTP1S that are important for different stages of OR trafficking, odorant-mediated responses, and interaction with ORs. However, the exact roles of RTP2 and the significance of the requirement of the seemingly redundant co-expression of the two RTP proteins *in vivo* have received less attention in the past. Here we attempted to dissect the functional differences between RTP1S and RTP2 using a HEK293T cell-based OR heterologous expression system. When a set of 24 ORs were tested against 28 cognate ligands, unlike RTP1S, which always showed a robust ability to support odorant-mediated responses, RTP2 had little or no effect on OR responses and exhibited a suppressive effect over that of RTP1S for a subset of the ORs tested. RTP1S and RTP2 showed no significant difference in OR ligand selectivity and co-transfection with RTP2 increased the detection threshold for some ORs. A protein-protein interaction analysis showed positive interactions among OR, RTP1S, and RTP2, corroborating the functional linkages among the three molecules. Finally, further cell-surface and permeabilized immunocytochemical studies revealed that OR and the co-expressed RTP1S proteins were retained in the Golgi when co-transfected with RTP2, indicating that RTP1S and RTP2 could play different roles in the OR trafficking process. By examining the functional differentiations between the two RTP family members, we provided a molecular level explanation to the suppressive effect exerted by RTP2, shedding light on the divergent mechanisms underlying the RTP proteins in regulating the functional expression of ORs.

## Introduction

Detecting and discriminating a large number of volatile chemicals is one of the essential survival skills for animals in nature. This ability is determined by the odorant receptors (OR) distributed at the ciliary surface of olfactory sensory neurons (OSN). Odorant receptor proteins constitute the largest family of the seven-transmembrane protein superfamily—G protein-coupled receptors (GPCR)—with 1194 members in the mouse genome and 387 members in the human genome [1–5]. Similar to some of the other GPCRs, precise trafficking of the OR proteins, involving the synthesis from the endoplasmic reticulum (ER) and the transport to the cell-surface membrane, is critical for OR function [6].

The elucidation of the functional mechanisms of ORs centers around the deorphanizing ORs for cognate ligands [7] that calls for the establishment of an efficient expression system to mimic OR functional expression in the OSNs in order to facilitate large-scale of screening of odorous chemicals. However, cultured cell lines of non-olfactory origins differ from native OSNs to the extent that OR proteins are retained in the ER and unable to be trafficked to the plasma membrane, resulting in OR degradation and loss of function [8, 9].

Extensive efforts have been made to achieve OR functional expression to the cell membrane in heterologous cell lines [6, 10–13]. There is evidence for homodimeric or heterodimeric interactions between GPCRs and for interactions between GPCRs and other transmembrane proteins, such as in the case of the Class B GPCR, calcitonin receptor-like receptor (CRLR), complexing with members of the receptor activity-modifying proteins (RAMPs) [14]. It is likely that there are interactive relationships between ORs and other RAMP-like chaperone proteins, which is absent in cultured cell lines and may enhance the function of ORs. Through a SAGE library analysis from single OSNs, Saito *et al*. first identified the receptor-transporting proteins, RTP1 and RTP2, that are specifically expressed in the OSNs, associate with OR proteins, and facilitate OR trafficking to the plasma membrane in human embryonic kidney (HEK) 293T cells [12]. A subsequent study found a shorter form of the RTP1 protein, RTP1S, that could more robustly promote ORs trafficking to the cell surface and more efficiently facilitate OR responses to cognate ligands compared to the original RTP1 protein [15]. Based on these findings, researchers have since established a high-throughput screening platform to match odorous ligands with the mammalian OR repertoire [16–22].

Like the RAMPs, RTP family members may have a general role in modulating GPCR trafficking and function. For example, co-expression of RTP3 and RTP4 could enhance the function of the human bitter taste receptor TAS2Rs [23]. Co-expression of RTP4 could increase the cell-surface expression of a heterodimer of two non-chemosensory GPCRs, the μ and δ opioid receptors [24]. To identify the domains of RTP1S involved in OR trafficking, we previously performed a structure-function analysis of RTP1S and found that RTP1S mediates the translocation and activation of ORs by acting through multiple steps. RTP1S could complex with OR proteins as an accessory protein during the whole OR trafficking process, including synthesis in the ER, transport to Golgi, and localization at the cell surface with its different functional domains. The transmembrane domain is especially critical for the localization of RTP1S to the lipid raft microdomain, which is essential for odorant mediated responses, hinting a more comprehensive role in OR functional expression for RTP family members [25].

With the roles of RTP1S thoroughly investigated, the function of RTP2 and the significance of the requirement of the coexistence of RTP1S and RTP2 *in vivo* have however received less attention. In this study, using a heterologous expression system, we examined the functional differences between RTP1S and RTP2 and the physiological significance of their coexistence. We found that RTP1S and RTP2 exerted different degrees of promotional effect on OR function and could play different roles in the OR trafficking process. Using immunocytochemistry, we proposed the molecular mechanisms underlying the divergent functions of two different RTP family members. These findings may promote the understanding of the working relationships among OR, RTP1S, and RTP2 in OSNs.

## Materials and methods

### Chemicals

The structures and sources of odorants used are described in Supplemental data (S1 Table). All chemicals were dissolved in DMSO or ethanol and diluted to 1 M stock solutions and kept at -20°C until use.

### Plasmid construction

Rho (MNGTEGPNFY-VPFSNATGVVR), FLAG (DYKDDDDK), and HA (MYPYDVPDYA) tags were subcloned into the pCI mammalian expression vector as described previously [15]. The open reading frames of ORs were amplified from mouse genomic DNA and subcloned into pCI expression vectors containing Rho or Flag tags. The RTP1S and RTP2 plasmid constructs were subcloned into pCI expression vectors containing Flag or HA tags. The sequences of all plasmid constructions were verified by sequencing.

### Cell culture

HEK293T cells were generously provided by Dr. Hiroaki Matsunami from Duke University Medical Center, USA, and were maintained in minimal essential medium (HyClone) containing 10% fetal bovine serum (Invitrogen), 500 μg/ml penicillin/streptomycin (HyClone), and 6 μg/ml amphotericin B (Sigma) at 37°C with 5% CO_2_.

### Luciferase assay and data analysis

The Dual-Glo^®^ luciferase assay system (Promega) was used for luciferase assay as described previously [25]. Two luciferase constructs were used, including a firefly luciferase gene driven by a 4x cAMP-response element (CRE-Luc) that was used to measure receptor activation and a *Renilla* luciferase gene driven by a constitutively active SV40 promoter (pRL-SV40; Promega) that was used as an internal control for cell viability and transfection efficiency. HEK293T cells were plated on poly-D-lysine-coated 96-well plates (Greiner). Plasmid DNAs of ORs and accessory proteins were transfected using Lipofectamine2000 (Invitrogen). For each 96-well plate, 1 μg of CRE-Luc, 1 μg of pRL-SV40, 0.5 μg of OR, and 1 μg total of all accessory proteins (RTP1S and/or RTP2) or pCI were transfected. For example, when 0.5 μg of each accessory protein was transfected alone, 0.5 μg of pCI was co-transfected to make the total amount of RTP constant. 16–18 hr post-transfection, the medium was replaced with CD293 chemically defined medium (Invitrogen) and then incubated for 30 min at 37 °C. The medium was then replaced with odorant solution diluted in CD293 and incubated for 4 hr at 37 °C. We followed the manufacturer’s protocols for measuring firefly luciferase (Luc) and *Renilla* luciferase (RL) activities. Luminescence was measured using Infinite F200 Pro plate reader (Tecan). Normalized luciferase activity was calculated by the formula [(luc/RL)_N_-(luc/RL)_min_]/ [(luc/RL)_max_-(luc/RL)_min_], where (luc/RL)_N_ = luminescence of firefly luciferase divided by luminescence of *Renilla* luciferase for a certain well; (luc/RL)_min_ = lowest luminescence of firefly luciferase divided by luminescence of *Renilla* luciferase of a plate or a set of plates; and (luc/RL)_max_ = highest luminescence of firefly luciferase divided by luminescence of *Renilla* luciferase of a plate or a set of plates. Data were analyzed with Microsoft Excel and GraphPad Prism 5.

### Interaction analysis

For a visualization of functional interactions induced by the combination of RTP1S and RTP2, we plotted the luciferase data on a synthetic graph [σ = f(τ)] introduced by Patte and Laffort [26]. In this equation, the parameter τ represents the ratio of the OR response level to cognate odorant diluted into a certain concentration when co-transfected with RTP1S or RTP2 to the algebraic sum of individual OR response level when co-transfected with single RTPs: τ_RTP1S_ = ψ_RTP1S_ / (ψ_RTP1S_ + ψ_RTP2_) or τ_RTP2_ = ψ_RTP2_ / (ψ_RTP1S_ + ψ_RTP2_) where ψ = OR response level expressed as the normalized luciferase value. Following this formula, τ_RTP1S_ + τ_RTP2_ = 1, and when τ = 0.5 (ψ_RTP1S_ = ψ_RTP2_), RTP1S and RTP2 can be considered as equally responsive in facilitating OR function. We qualified the functional interactions induced by the co-expression of RTP1S and RTP2 by computing the parameter σ representing the ratio of OR response level when co-expressed with the mixture of RTP1S and RTP2 (ψ_mix_) to the algebraic sum of individual OR response level when co-transfected with single RTPs: σ = ψ_mix_ / (ψ_RTP1S_ + ψ_RTP2_). Following this formula, the interactions between the co-expressed RTP1S and RTP2 for OR function can be qualified into three divisions: (1) hyper-addition when σ > 1, representing a synergistic effect between the co-expressed RTP1S and RTP2 in facilitating OR function; (2) complete addition when σ = 1, representing the OR response level elicited by the co-expression of RTP1S and RTP2 equals exactly to the sum of individual OR response level when co-transfected with single RTPs; (3) hypo-addition when σ < 1, representing a competitive effect between the co-expressed RTP1S and RTP2 in facilitating OR function. Based on the computed σ and τ_RTP1S_, a scatter diagram can be plotted to represent the functional interactions induced by the combination of RTP1S and RTP2. In a plot where [σ = f(τ_RTP1S_)], the OR activation is mainly promoted by RTP1S when the scatters aggregate on the right side of the diagram (where τ_RTP1S_ = 1); in contrast, OR activation is mainly promoted by RTP2 when the scatters are seen on the left side of the diagram (where τ_RTP1S_ = 0, τ_RTP2_ = 1). The spacial divisions segmented by the diagonals in the diagram reflect the relationships among ψ_RTP1S_, ψ_RTP2_, and ψ_mix_ (S1 Fig).

### Immunoprecipitation

HEK293T cells in 60-mm dishes were transfected with N-terminal Flag-tagged OR and/or C-terminal HA-tagged/Flag-tagged RTPs. 16 hr after transfection, cells were lysed with lysis buffer (50 mM Tris [pH 7.4], 150 mM NaCl, 1% NP-40, 0.5 mM PMSF, 1% protease inhibitor cocktail). The lysates were incubated with anti-Flag M2 affinity gel (Sigma) or anti-HA affinity matrix (Roche) overnight at 4 °C and washed with lysis buffer. The bound proteins were eluted by incubation with 1x SDS sample buffer at room temperature for 2 hr and -80 °C overnight and then subjected to Western blot.

### Western blot

Proteins from immunoprecipitation were resolved by sodium dodecyl sulfatepolyacrylamide gel electrophoresis using a mini gel apparatus (Bio-Rad) and subsequently electrophoretically transferred onto nitrocellulose membranes. The membranes were blocked with a blocking solution (Tris-buffered saline with 5% non-fat milk and 0.1% Tween-20) for 2 hr at room temperature, incubated with the primary antibodies, rabbit anti-HA or rabbit anti-Flag, dissolved in blocking solution overnight at 4 °C, and finally with horseradish peroxidase-conjugated secondary antibodies (Beyotime) dissolved in blocking solution for 2 hr at room temperature. The signals were detected by Immobilon Western Chemiluminescent HRP Substrate (Millipore) according to the manufacturer’s instructions.

### Chemiluminescent immunoassay for OR cell-surface expression

HEK293T cells were plated on poly-D-lysine-coated 96-well plates (Greiner). Plasmid DNA of ORs containing Rho tag and accessory proteins were transfected using Lipofectamine2000 (Invitrogen). For each 96-well plate, 3.2 μg of OR and 6.4 μg total of all accessory proteins (RTP1S and/or RTP2) or pCI vector as a negative control were transfected. For example, 3.2 μg of one accessory protein and 3.2 μg of pCI were transfected to make the total amount of plasmids constant. 16–18 hr post-transfection, cells were fixed in 4% paraformaldehyde for 15 min and blocked in 5% FBS/0.5% BSA diluted in phosphate-buffered saline at 4 °C. The cells were then incubated in 5% FBS/0.5% BSA diluted in phosphate-buffered saline containing the primary antibody mouse anti-rhodopsin (Millipore) at 4 °C for 1 hr. The cells were then washed in phosphate-buffered saline followed by incubation with horseradish peroxidase-conjugated secondary antibodies (Beyotime) at 4 °C for 45 min. The signals were detected by Immobilon Western Chemiluminescent HRP Substrate (Millipore) according to the manufacturer’s instructions and luminescence was measured using the Infinite F200 Pro plate reader (Tecan). Normalized luminescence values for quantifying the OR cell-surface expression were calculated by the formula OR/pCI. Data were analyzed with Microsoft Excel and GraphPad Prism 5.

### Immunocytochemistry

Live-cell surface staining was performed as described previously [15]. For all staining, 0.8 μg of OR DNA and 0.8 μg total of all RTP proteins (RTP1S and/or RTP2) or pCI were transfected per dish. 0.3 μg of green fluorescent protein (GFP) was transfected as a control for transfection efficiency. The primary antibodies used were mouse anti-rhodopsin (Millipore), rabbit anti-HA (Sigma), and rabbit anti-FLAG (Sigma). The secondary antibodies used were Alex Fluor 555- or 488-conjugated anti-rabbit and anti-mouse IgG (Invitrogen). Permeabilized staining was performed as described previously [25]. The cells were permeabilized with methanol at 4 °C for 5 min after fixation. Anti-calnexin antibody (Abcam) was used for ER staining. For Golgi staining, cells were incubated with Alex Fluor 488-conjugated wheat germ agglutinin (Invitrogen) for 20 min followed by incubation with a secondary antibody. For all staining, slides were mounted with VectaShield (Vector) and visualized by a fluorescent microscope (ZEISS Imager A2). To quantify the percentage of ORs or RTPs colocalization with the ER or Golgi apparatus, cells were doubly stained with the respective epitope tags for ORs or RTPs (Alex Fluor 555) and for ER or Golgi markers (Alex Fluor 488). As assessed by a co-transfected GFP plasmid, we estimated the transfection efficiency of the system to be ~ 40%; thus, on each slide, we counted 100 cells during each counting session with Alex Fluor 555 signals and subsequently recorded the number of cells in which Alex Fluor 555 and Alex Fluor 488 overlapped. To quantify the percentages of cells that were expressed on the cell-surface, we co-transfected the cells with GFP, counted 100 cells with GFP during each counting session, and recorded the number of cells that had punctate cell-surface signals.

### Fluorescence-activated cell sorter (FACS) analysis

FACS analysis was performed as described previously [15]. Briefly, HEK293T cells were seeded in 35-mm dishes and then transfected with the same amount of plasmid DNA as used for immunocytochemistry. 2 ng of GFP was transfected per dish as a control for transfection efficiency. 24 hr post-transfection, the cells were dissociated with Cellstripper^TM^ (Corning Cellgro) and transferred to a tube for incubation with the anti-rhodopsin antibody as described for immunocytochemistry and then with phycoerythrin-conjugated donkey anti-mouse IgG (Jackson ImmunoResearch Laboratories, Inc.). Fluorescence was analyzed using a FACSCalibur (BD Biosciences).

## Results

### RTP1S and RTP2 differ in their abilities to promote the functional activation of ORs

**Fig 1:**
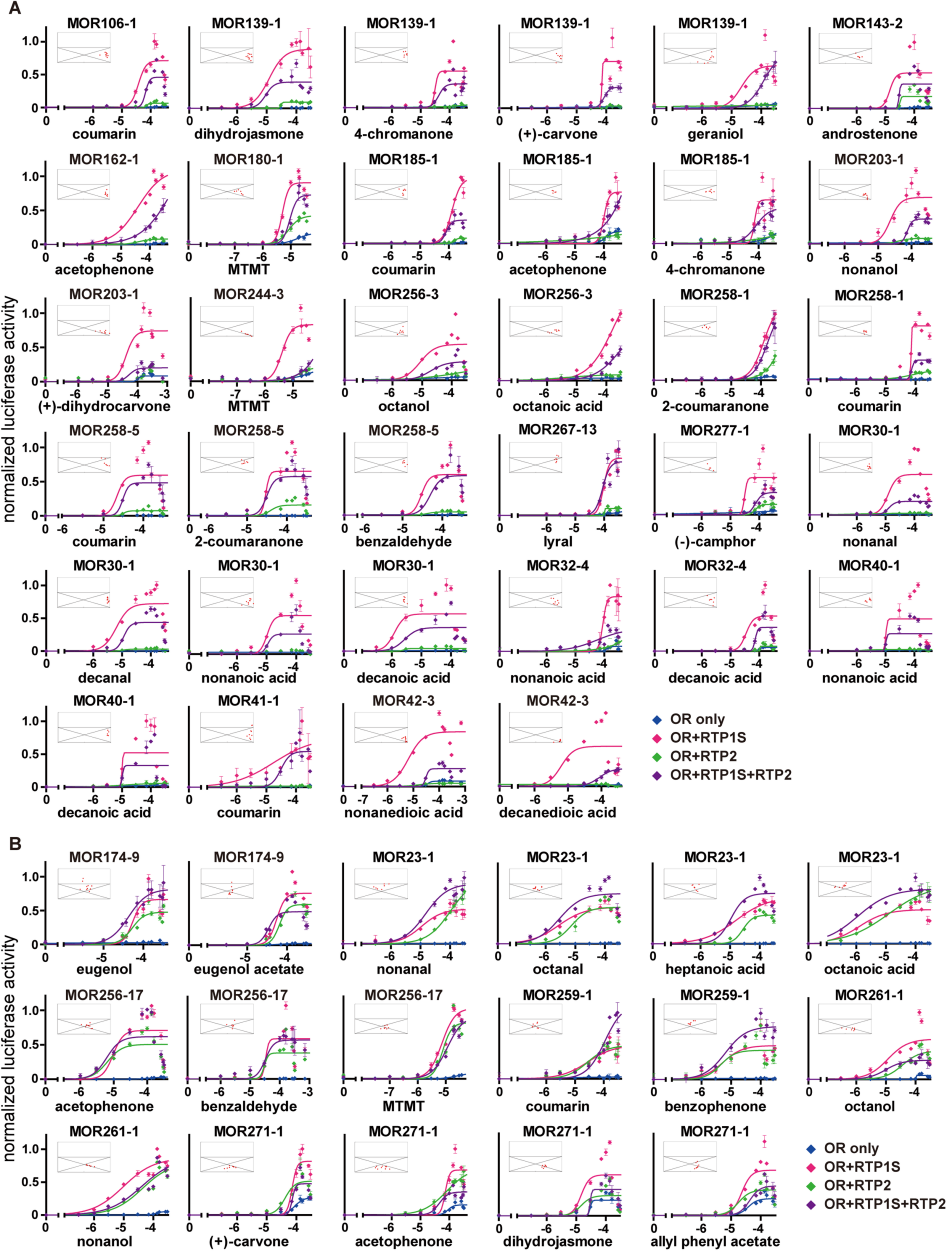
RTP1S and RTP2 differ in their abilities to promote the functional activation of ORs. (A-B) normalized luciferase activities of concentration gradients of 28 odorants from 0 μM to 30, 300 or 320 μM tested against 24 ORs with different combinations of RTPs co-transfected, including RTP1S (red), RTP2 (green), and a combination of the two (purple) in HEK293T cells. An “OR only” negative control is co-transfected with the empty pCI vector (blue). The *x*-axis represents molar odorant concentrations on a logarithmic scale. The *y*-axis represents normalized luciferase activity shown as mean ± S.E.M. (*N* = 3). The scatter diagrams (inset) depict the functional effect induced by the combination of RTP1S and RTP2 compared to RTP1S or RTP2 alone (*Materials and methods* and S1 Fig). The *x*-axis represents the value of the parameter τ_RTP1S_, which represents the ratio of the OR response level when co-transfected with RTP1S to the algebraic sum of individual OR response levels when co-transfected with single RTPs. The *y*-axis represents the value of the parameter σ, with σ = 1 dividing the diagram into hyper-addition and hypo-addition sections. The responses at lower concentrations that did not elicit OR activation were not plotted.

To investigate the functional differences between RTP1S and RTP2, we first employed a luciferase reporter gene assay to quantify the activation of ORs by their ligands when co-transfected with different RTPs or a combination of the two RTPs in HEK293T cells. We tested a total of 28 odorants against 24 ORs, representing a total of 51 receptor ligand pairs. We found that while co-expression of ORs with RTP1S alone could markedly enhance odorant-dependent luciferase activities, as previously shown (12,15), co-expression with RTP2 showed significantly smaller enhancements and had little or no effect for 34 of the receptor-ligand pairs. Moreover, in these pairs, co-transfection with a combination of RTP1S and RTP2 showed a lower level of response compared to RTP1S alone, indicating a suppressive effect of RTP2 when co-expressed with RTP1S (Fig 1A). In the other 17 receptor-ligand pairs, representing 16 odorants tested against 6 ORs, RTP2 showed a stronger ability in improving OR responses compared to the pairs shown in Fig 1A. Though RTP2 still showed weaker effect on ORs than RTP1S, the negative effect of RTP2 appeared to be less obvious when RTP1S and RTP2 were co-expressed (Fig 1B). Unlike the general promotional effect of RTP1S for all screened ORs, only the 6 ORs in Fig 1B benefited from the expression of RTP2. Moreover, for the activations of all 24 ORs, the promotional effect of the combination of the two RTPs does not equal the simple sum of the effects of each of the RTPs.

To characterize the functional relationships between the two RTPs, we further represented the luciferase data on scatter diagrams (*Materials and methods* and S1 Fig) shown as the insets in Fig 1. We found that co-expression of RTP1S and RTP2 elicited hypo-additive effects in the 51 receptor-ligand pairs tested since all the scatters of these response pairs aggregated in the divisions where the parameter σ < 1. We attributed these hypo-additive effect to the possibility that the two RTP proteins with different efficacy for OR function may share the main binding site on a given OR protein, resulting in a diminishing effect between the co-expressed RTP1S and RTP2 on OR activation. The scatters of the response pairs in Fig 1A gathered on the right side of the diagrams (where τ_RTP1S_ = 1), meaning that the activations of these ORs mainly depended on the promotional effects of RTP1S, whereas the scatters mainly gathered toward the middle (where τ_RTP1S_ = 0.5) in Fig 1B, indicating both of the two RTPs exhibited similar enhancement effects for the activations of these ORs. By replotting these scatter diagrams for 16 ORs against different cognate ligands and for 14 odorants against different types of ORs, we found that the mode of interaction between the two RTPs depended more on OR type than odorant type (S2 Fig).

Based on the above results, we classified the screened ORs into two categories. In Category 1 (18 ORs represented in Fig 1A: MOR106-1, MOR139-1, MOR143-2, MOR162-1, MOR180-1, MOR185-1, MOR203-1, MOR244-3, MOR256-3, MOR258-1, MOR258-5, MOR267-13, MOR277-1, MOR30-1, MOR32-4, MOR40-1, MOR41-1, and MOR42-3), the function of RTP1S was much more prominent than RTP2 and RTP2 had a suppressive effect on RTP1S. In Category 2 (6 ORs represented in Fig 1B: MOR174-9, MOR23-1, MOR256-17, MOR259-1, MOR261-1, and MOR271-1), both RTPs could enhance OR response levels and RTP2 does not decrease the function of RTP1S.

### RTP1S and RTP2 show no significant difference in OR ligand selectivity

**Fig 2:**
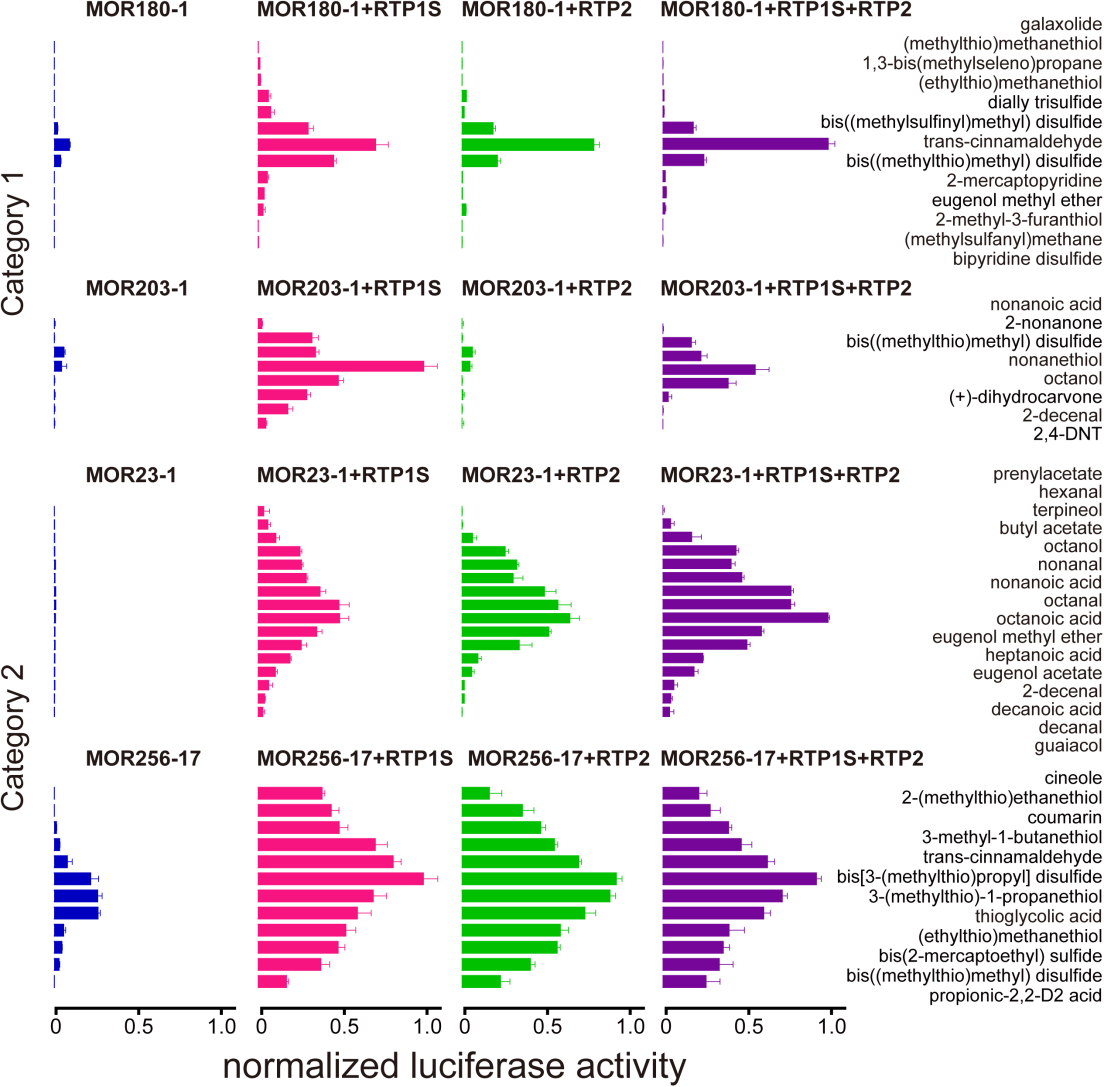
RTP1S and RTP2 show no significant difference in OR ligand selectivity. Normalized luciferase activities of 4 representative ORs from Category 1 (MOR180-1 and MOR203-1) and Category 2 (MOR23-1 and MOR256-17), transfected alone (blue) or co-transfected with different combinations of RTPs, including RTP1S (red), RTP2 (green), and a combination of the two (purple), and tested against various ligands at a fixed concentration of 100 μM. The *x*-axis represents normalized luciferase activity shown as mean ± S.E.M. (*N* = 6).

We next chose two ORs from each of the two OR categories and each of the ORs was tested against a panel of ligands to see whether different RTPs would affect their ligand selectivity (Fig 2). For MOR180-1 and MOR203-1 from Category 1, the existence of RTP2 decreased the numbers of ligands at the tested concentration (100 μM), supportive of a suppressive role of RTP2, while, for MOR23-1 and MOR256-17 from Category 2, co-expression with RTP1S or with RTP2 showed no significant difference in the numbers of responsive ligands (Fig 2). We then tested two higher concentrations (300 and 1000 μM) of the same subset of ligands for one receptor from each of the two OR categories (S3 Fig). We found that for the Category 1 OR, MOR203-1, when co-transfected with RTP2, increasing the ligand concentration can restore the responses of the OR for most of its ligands, indicating that the presence of RTP2 may increase the OR’s detection threshold by shifting the response curves to the right.

### OR, RTP1S, and RTP2 can interact with each other in HEK293T cells

**Fig 3:**
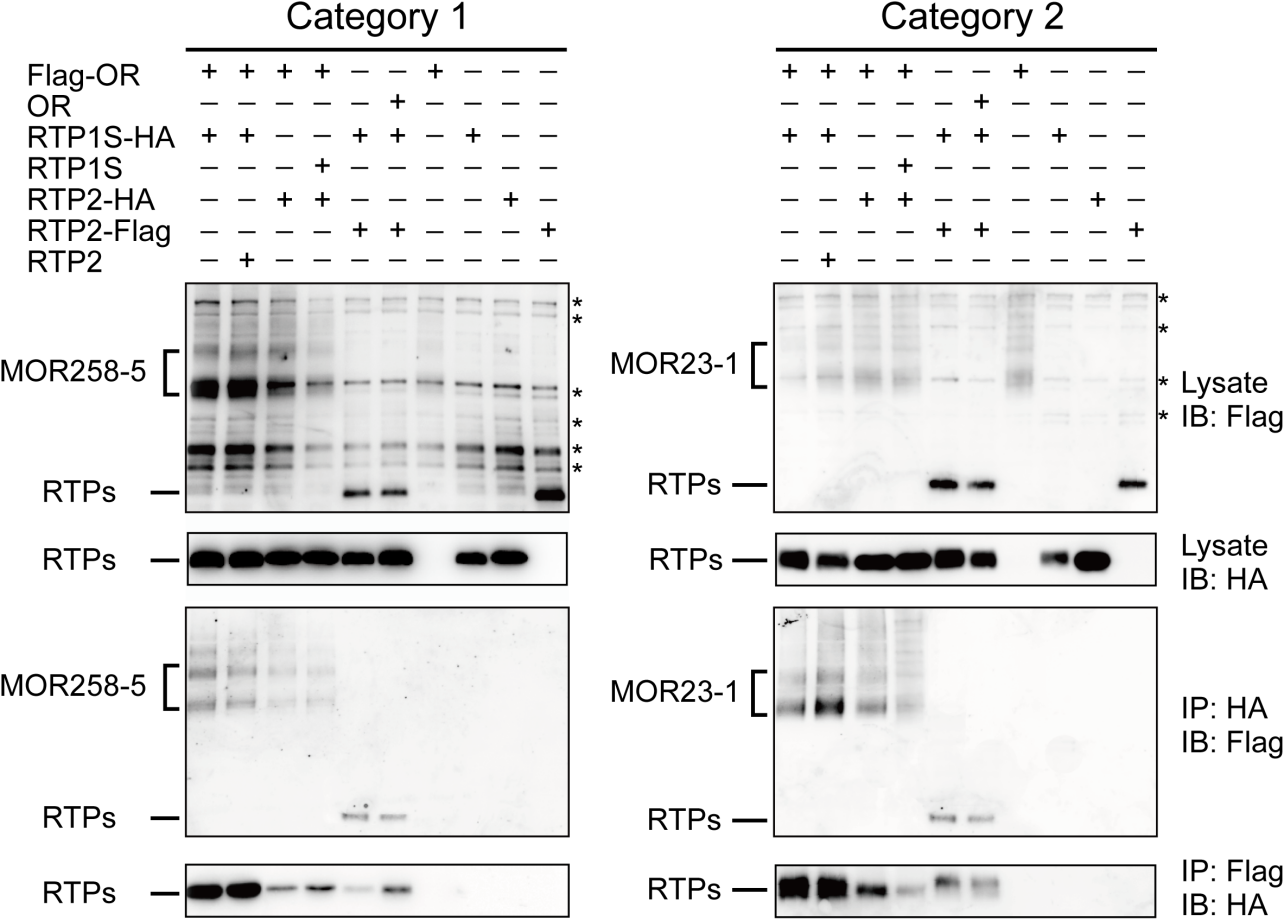
OR, RTP1S, and RTP2 interact with each other in HEK293T cells. *Left*, interactions among MOR258-5 (Category 1), RTP1S and RTP2. *Right*, interactions among MOR23-1 (Category 2), RTP1S and RTP2. *First and second panels*, protein lysates of HEK293T cells transfected with Flag-tagged OR and/or HA-tagged RTP1S and Flag-tagged/HA-tagged RTP2 and blotted with anti-Flag or anti-HA antibody. *Third panels*, co-immunoprecipitation of Flag-tagged proteins with anti-HA antibody. *Fourth panels*, co-immunoprecipitation of HA-tagged proteins with anti-Flag antibody. Refer to S4 Fig for the original blots. *IB*, immunoblot; *IP*, immunoprecipitation. The asterisks indicate non-specific bands.

The functional linkages among ORs and the two RTPs indicated possible direct protein-protein interactions. Previously, it was shown that different OR proteins could interact with RTP1/RTP1S [12, 25]. We next investigated whether further interaction existed between OR and RTP2, and between RTP1S and RTP2, using one typical OR from each of the two categories (MOR258-5 from Category 1 and MOR23-1 from Category 2). We found that regardless of the type of OR used, OR always co-immunoprecipitated with RTP1S or RTP2, and *vice versa*. In addition, RTP1S and RTP2 co-immunoprecipitated with each other (Fig 3).

### RTP2 played a suppressive role in OR cell-surface expression when co-expressed with RTP1S

Given the aforementioned differences between RTP1S and RTP2 in the promotion of receptor function and to directly compare and contrast the functions of RTP1S and RTP2 in OR trafficking, we next analyzed the effect of the different RTP proteins on the cell-surface expression of 14 different types of ORs that are typically expressed on the cell surface using a high-throughput chemiluminescent quantification assay. All of the screened ORs (9 from Category 1 and 5 from Category 2) could be transported to the cell surface by RTPs or on their own. RTP1S could significantly promote the trafficking of these ORs to the cell surface; however, regardless of OR category, the ability of RTP2 was limited compared to RTP1S, and the function of RTP1S was decreased when RTP2 was co-transfected (S5 Fig).

To confirm these results, we compared the cell-surface expression of the 4 selected ORs from Fig 2 using live-cell staining. As expected, we observed far more immunofluorescent cells and stronger signals when the ORs were transfected with RTP1S compared to when expressed alone or when transfected with RTP2, which apparently had limited effect on the promotion of OR cell-surface expression, consistent with a previous report [12]. In addition, the promotional effect of OR cell-surface expression by RTP1S were decreased when RTP2 were co-expressed, again indicating a suppressive role of RTP2 in OR cell-surface expression (S6A Fig). A follow-up quantification experiment using FACS confirmed that RTP1S was usually the most potent accessory protein in promoting the cell-surface expression of ORs compared with RTP2 or the co-expression of RTP1S and RTP2 (S6B Fig).

### RTP1S and RTP2 play divergent roles in OR trafficking

**Fig 4:**
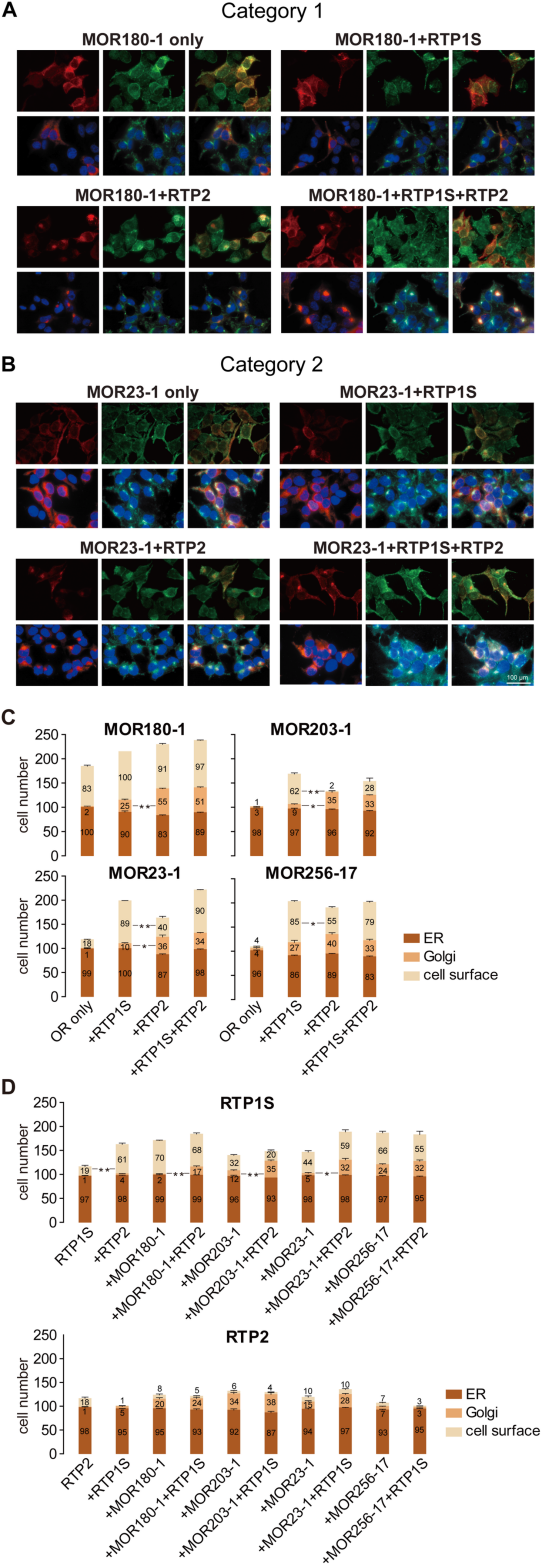
RTP1S and RTP2 play divergent roles in OR trafficking. (A-B) permeabilized staining were performed to examine the subcellular localizations of MOR180-1 (Category 1) and MOR23-1 (Category 2) when they were transfected alone or cotransfected with different RTPs or a combination of the two RTPs. *Left panels*, red signals represent the localization of OR proteins. *Middle panel*, green signals represent staining of organelles; *first row*, staining of ER with calnexin; *second row*, staining of Golgi with wheat germ agglutinin. *Right panels*, yellow signals represent OR proteins merged with the corresponding organelle. Blue signals in the *second row* are DAPI nuclear staining. Scale bar, 100 μm. (C-D) quantification of the cell-surface expression and subcellular localizations of the 4 tested ORs and RTPs. Columns represent the cells of each counting session where the OR or RTP proteins were localized in the ER, the Golgi, or at the cell surface. The *y*-axis represents the cell numbers of each counting session where the OR or RTP proteins were localized in ER, Golgi, or at the cell surface, shown as mean ± S.E.M. (*N* = 3). Paired two-tailed *t* test was used to compare the localizations in certain organelle or at the cell surface when different combinations of OR and RTP were cotransfected. * *P* < 0.05, ** *P* < 0.01.

Since RTP1S and RTP2 each seemed to promote the cell-surface expression of ORs to a different degree, we next investigated the roles of RTP1S and RTP2 in the OR trafficking process by examining the subcellular localizations of ORs from both of the two categories and RTPs in HEK293T cells. We used both live-cell staining to detect the cell-surface expression of ORs and permeabilized staining to detect the subcellular localization of ORs with the markers for ER and the Golgi apparatus. As previously reported [25], both MOR180-1 (Category 1) and MOR23-1 (Category 2) colocalized with the ER marker rather than the Golgi marker when transfected on their own. However, when co-transfected with each of the two different RTPs or a combination of the two, the OR proteins exhibited significant variations in subcellular localizations. For both MOR180-1 and MOR23-1, compared to when they were transfected alone, we observed decreased OR signals in the ER and increased OR signals colocalizing with the Golgi marker when they were co-transfected with RTP1S. This may indicate that a large proportion of the OR proteins are transported to the membrane by RTP1S via the Golgi apparatus. Interestingly, consistent with the fact that RTP2 exhibited very limited effect on the promotion of ORs to the cell surface and that it may even counteract the function of RTP1S when the two RTPs coexisted, permeabilized staining showed much stronger MOR180-1 and MOR23-1 signals being retained in the Golgi when the OR proteins were co-expressed with RTP2. Similar OR retentions in the Golgi apparatus were also observed when ORs and the two RTPs coexisted (Fig 4A and 4B). These results indicated that RTP2 may be more potent in transporting ORs from the ER to the Golgi apparatus than transporting ORs from the Golgi to the cell membrane. Similar localization patterns were also seen in the other two types of ORs: MOR203-1 (Category 1) and MOR256-17 (Category 2) (S7 Fig). Statistical analysis of the cell-surface and subcellular localizations of these 4 ORs revealed that, compared to RTP1S, RTP2 retained more OR proteins in the Golgi, which resulted in less OR proteins transported from the Golgi to the cell surface (Fig 4C and S2 Table). These results provide a molecular level explanation for the functional differentiation between the two RTPs and the suppressive role of RTP2 in OR function and expression.

To further dissect the roles of RTP1S and RTP2 in OR trafficking, we also analyzed the cell-surface and subcellular localizations of the co-transfected RTPs (Fig 4D and S3 Table). Most of the RTP1S and RTP2 proteins were localized in the ER when transfected on their own. The localizations of RTP1S were significantly altered when co-transfected with RTP2, with ORs, or with the combination of the two. RTP1S showed similar localization patterns with the ORs in Fig 4C when they were co-transfected, reinforcing a reciprocal trafficking of the two molecules as previously reported [25]. Moreover, co-transfection with RTP2 could promote RTP1S trafficking to the cell surface and the existence of RTP2 in the co-transfection system could retain more RTP1S proteins in the Golgi, confirming the interactions between RTP1S and RTP2. However, unlike RTP1S, RTP2 was unable to be transported from the Golgi to the cell surface no matter they were co-transfected with RTP1S, with ORs or with the combination of the two. An exception was seen in the case of MOR256-17 where RTP2 could not even be transported to the Golgi, which resulted less obvious Golgi retentions of the co-transfected MOR256-17 and RTP1S in Fig 4C and 4D.

## Discussion

An understanding of how GPCR accessory proteins regulate the localization and function of GPCRs is essential for resolving the molecular mechanisms of GPCR function. In this study, we thoroughly investigated the functional differentiation between the two RTP family members, RTP1S and RTP2, which are specifically expressed in OSNs and facilitate OR function. We found that the ability of RTP1S to promote OR function was in absolute advantage over RTP2, which had little or no effect for 34 receptor-ligand pairs representing 18 types of ORs tested against 22 odorants. Interestingly, in these pairs, cotransfection of both RTP1S and RTP2 showed a lower level of response compared to RTP1S alone, indicating a suppressive role of RTP2. In addition, we isolated another set of 17 receptor-ligand pairs, representing 6 types of ORs tested against 16 odorants, where both RTP1S and RTP2 could significantly promote their response levels and the suppressive role of RTP2 was less obvious. Further analysis of the 51 pairs revealed the different functional relationships between the two RTPs depended more on OR type than odorant type. Based on these findings, we classified the 18 ORs represented in the 34 response pairs as Category 1 ORs, and the other 6 ORs in the 17 response pairs as Category 2 ORs. Furthermore, we found that the coexistence of the two RTPs always elicited hypo-additive effects on all of the screened receptor-ligand pairs, pointing to a possible competitive interaction between the two RTPs. Co-immunoprecipitation data also supported protein-protein interactions among ORs, RTP1S, and RTP2.

We also compared the differences between RTP1S and RTP2 in altering OR ligand selectivity. Unlike the RAMPs, coupling with RTP1S or RTP2 showed no significant difference in ligand selectivity for ORs. However, the detection thresholds of ORs from Category 1 were increased when co-transfected with RTP2 or with the combination of the two RTPs, recapitulating the suppressive role of RTP2. Compared to the widely promotional effect of RTP1S for all types of tested ORs, co-expression with RTP2 only benefited a limited number of OR types in odorant-mediated activations. Since the extracellular domains of RTP1S and RTP2 are very short, it may be unlikely that they participate in the OR-ligand binding process, but they may modulate OR activation through orthosteric or allosteric mechanisms that may eventually contribute to downstream signaling transduction variations. Despite the seemingly negative role of RTP2 seen so far, one possibility is that the modulatory effect of RTP2 on ORs could serve as a part of the checks and balances system *in vivo* to curtail the levels of OR responses.

There are several barriers along the secretory pathway to the cell membrane for ORs to overcome, including correct protein folding in the ER, successful ER exit, transportation through the Golgi, and vesicular transportation to the membrane [6]. Previous studies showed reciprocal expression of ORs with RTP1S and RTP1S could complex with ORs with its different functional domains during the whole OR trafficking process. The 17 amino acid residues in the N-terminal domain of RTP1S are important for transporting ORs exit from the ER, and a middle region of around 80 amino acids of RTP1S is responsible for the OR to pass through the Golgi [12, 15, 25]. Here, we found distinct roles of RTP1S and RTP2 in facilitating OR trafficking to cell membrane. Unlike the role of RTP1S in targeting the ORs to the cell surface, RTP2 might be more involved in transporting ORs from the ER to the Golgi apparatus than transporting ORs from the Golgi to the cell membrane, as corroborated by the strong OR signals that are restricted to the Golgi when cotransfected with RTP2, the limited promotional effect of RTP2 in facilitating OR cell-surface expression, and the absence of RTP2 localization on the cell surface. Although the function of RTP2 is different for ORs from the two different categories, there is no obvious functional division of the two RTPs in the trafficking processes for different types of ORs from different categories. Furthermore, an examination of the protein sequence alignment of the middle segment putative for Golgi exit previously identified through the use of RTP1S/RTP4 chimeras [25] revealed 11 amino acid residues that are not conserved between RTP1S and RTP2; among these, 2 amino acid residues are either conserved or more similar in side chain properties between RTP2 and RTP4, and 7 other amino acid residues are different among RTP1S, RTP2, and RTP4 (S8 Fig). Future mutational studies of RTP2 may reveal residues key to its Golgi retentional property.

In addition, we also found that RTP2 might significantly affect RTP1S in the transportation process. Firstly, far more immunofluorescent cells and stronger cell-surface signals of RTP1S were seen when RTP2 was cotransfected, indicating a possible promotional effect of RTP2 in transporting RTP1S to the cell surface. However, this effect seemed to be unreciprocated since the localization of the cotransfected RTP2 on the cell surface was still absent. Moreover, RTP2 retained more RTP1S proteins in the Golgi apparatus when OR proteins were cotransfected with the two RTPs. As for the localizations of RTP2 cotransfected with ORs or/and RTP1S, more RTP2 signals were colocalized with the Golgi marker compared to when transfected alone, reconfirming an important role of RTP2 in transporting the coexpressed proteins from the ER to Golgi. These results are indicative of direct interactions among ORs, RTP1S, and RTP2 along the secretory pathway. The localization patterns could be seen in all of the screened ORs from both of the two categories, with the exception of MOR256-17 (Category 2), in which cotransfection with RTP2 showed no significant differences in the Golgi apparatus localizations for MOR256-17 and RTP1S and the cotransfected RTP2 was retained in the ER rather than localized in Golgi or at the cell surface.

It is questionable whether the odorant-mediated responses of ORs are always concordant with their cell surface expressions. For example, MOR143-2 is unable to be expressed on the cell-surface membrane of HEK293T cells with or without accessory proteins, however, the downstream cAMP signaling pathway could still be activated by its ligand androstenone with the coexpression of RTPs. Similarly, MOR162-1, MOR267-13, MOR32-4, MOR40-1, and MOR174-9 all lacked cell-surface expression, but all could be activated by their cogante ligands (S9 Fig). This indicates that the odorant-mediated responses of some ORs may not necessarily depend on their targeting to the cell membrane. Numerous GPCRs have been verified to localize to intracellular membranes such as nuclear and mitochondrial membranes in different primary cell systems, including cardiac myocytes, endothelial cells, vascular smooth muscle cells, neurons, hepatocytes and kidney cells [27–29]. Hydrophobic ligands, such as prostaglandins, can cross membranes to access the intracellular receptors. However, hydrophilic ligands require the presence of some form of active or passive transport mechanism to cross the plasma membranes. Furthermore, a recent study on the functions of the intracellular GPCRs found that a cell-permeable ligand could access intracellular angiotensin receptors, resulting in an increased level of activation of the intracellular receptor compared to the same receptor located on the cell-surface membrane [30]. As for the intracellular ORs, it is possible that some odorants with small molecular weight could penetrate the plasma membrane and initiate the ORs responses at cytoplasmic membranes. The exact role and mechanism of the intracellular ORs remains to be investigated.

In summary, our study found that RTP2 exerts a suppressive effect over RTP1S, and RTP1S and RTP2 play different roles in the OR trafficking process, all pointing to divergent mechanisms underlying the function of the two RTP family members in association with ORs. We note that our study is based on an OR heterologous expression system, which may not entirely replicate the *in vivo* situation. A close examination of recently publilshed single OSN RNA sequencing data shows that among a total of 109 mature OSNs sequenced, RTP1S and RTP2 are always co-expressed alongside an OR [31–33]. Finally, a recent study utilizing the RTP1 and RTP2 double-knockout mice effectively analyzed the functional impacts of the two chaperone proteins *in vivo*. It was found that ORs could not be transported to the plasma membrane in the OSNs of the double-knockout mice, leading to decreased OSN activation and ultimately affecting OR gene choice [34].

## Supporting information

S1 Fig A scatter diagram for the interaction analysis of RTP1S and RTP2. The *x*-axis represents the value of the parameter τ_RTP1S_, which equals to ψ_RTP1S_ / (ψ_RTP1S_ + ψ_RTP2_). The *y*-axis represents the value of the parameter σ, which equals to ψ_mix_ / (ψ_RTP1S_ + ψ_RTP2_). ψ_RTP1S_, ψ_RTP2_, and ψ_mix_ equal to the OR response levels to ligands when co-transfected with RTP1S, RTP2, or the combination of the two, respectively. The combination of RTP1S and RTP2 induced three types of functional interactions, hyper-addition when σ > 1, complete addition when σ = 1, and hypo-addition when σ < 1. The spaces separated by diagonals in the hypo-addition section reflect the relationships among ψ_RTP1S_, ψ_RTP2_, and ψ_mix_.
(TIF)

S2 Fig The functional interaction between RTP1S and RTP2 depends more on OR type than odorant type. (A) characterization of the functional effect induced by the combination of RTP1S and RTP2 as compared to RTP1S or RTP2 alone for 16 ORs tested against various odorants. The overlaying spatial positions in each of the inset scatter diagrams represent the functional interactions between RTP1S andRTP2 for a certain OR against different odorants. (B) characterization of the functional effect induced by the combination of RTP1S and RTP2 as compared to RTP1S or RTP2 alone for 14 odorants tested against different types of ORs. The overlaying spatial positions in each of the inset scatter diagrams represent the functional interactions between RTP1S and RTP2 for a certain odorant tested against different types of ORs. See Fig 1 for the original dose-response curves.
(TIF)

S3 Fig Response levels of representative ORs to their cognate ligands in high concentrations. Normalized luciferase activities of representative ORs from Category 1 (MOR203-1) and Category 2 (MOR256-17), transfected alone (I) or co-transfected with different combinations of RTPs, including RTP1S (II), RTP2 (III), and a combination of the two (IV), and tested against the same subsets of ligands as in Fig 2. Different shades of grey columns represent ligand concentrations (0, 100, 300, and 1000 μM). The *y*-axis represents normalized luciferase activity shown as mean ± S.E.M. (*N* = 3).
(TIF)

S4 Fig Original blots for the co-immunoprecipitation results in Fig 3. (A) co-immunoprecipitation among MOR258-5 (Category 1), RTP1S, and RTP2. *Left panels*, protein lysates of HEK293T cells transfected with Flag-tagged MOR258-5 and/or HA-tagged RTP1S and Flag-tagged/HA tagged RTP2; *middle panels*, co-immunoprecipitation of HA-tagged proteins with anti-Flag antibody; *right panels*, co-immunoprecipitation of Flag-tagged proteins with anti-HA antibody. (B) co-immunoprecipitation among MOR23-1 (Category 2), RTP1S, and RTP2. *Left panels*, protein lysates of HEK293T cells transfected with Flag-tagged MOR23-1 and/or HA-tagged RTP1S and Flag-tagged/HA-tagged RTP2; *middle panels*, co-immunoprecipitation of HA-tagged proteins with anti-Flag antibody; *right panels*, co-immunoprecipitation of Flag-tagged proteins with anti-HA antibody. The asterisks indicate non-specific bands.
(TIF)

S5 Fig RTP2 exerts a suppressive role in OR cell-surface expression when co-expressed with RTP1S. Normalized cell-surface expression quantification of 14 ORs cotransfected with or without different combinations of RTP members including RTP1S (red), RTP2 (green), and a combination of the two (purple). An “OR only” negative control is cotransfected with the empty pCI vector (blue). Transfection with the vector pCI was used as a control divided by all of the read-out values. The grey dotted line represents an arbitrary minimum threshold value for determining cell-surface expression. The *y*-axis represents normalized luminescence value shown as mean ± S.E.M. (*N* = 3).
(TIF)

S6 Fig A suppressive role of RTP2 in OR cell-surface expression when co-expressed with RTP1S. (A) 4 representative ORs from both of the two categories (MOR180-1, MOR203-1, MOR23-1 and MOR256-17) were transfected with or without the accessory proteins RTP1S, RTP2, or the combination of the two in HEK293T cells. Cell-surface fluorescent OR signals are seen as punctate staining. Scale bar, 100 μm. (B) flow cytometry analysis of the cell-surface expression of the 4 ORs with or without the accessory proteins. Transfection with the empty pCI vector was used as a negative control. The intensity of phycoerythrin (PE) signal among the GFP-positive population was measured and plotted.
(TIF)

S7 Fig RTP1S and RTP2 play divergent roles in OR trafficking. (A-B) subcellular localization of MOR203-1 (Category 1) and MOR256-17 (Category 2) when they were transfected alone or co-transfected with different RTPs or a combination of the two RTPs. *Left panels*, red signals represent the localization of OR proteins. *Middle panels*, green signals represent staining of organelles; *first row*, staining of ER; *second row*, staining of Golgi. *Right panels*, yellow signals represent OR proteins merged with the corresponding organelle. Blue signals in the second row are DAPI nuclear staining. Scale bar, 100 μm.
(TIF)

S8 Fig An amino acid sequence alignment of RTP1S, RTP2, and RTP4. Intracellular, transmembrane, and extracellular domains are delimited with red vertical lines. The residues shaded in grey are conserved between RTP1S and RTP2 (74% identity). The green line spans residues 38 to 117 of RTP1S, representing the middle segment putative for Golgi exit. In this segment, residues shaded in purple are conserved or similar in the side chain properties between RTP2 and RTP4, and residues shaded in yellow are different among RTP1S, RTP2, and RTP4.
(TIF)

S9 Fig Normalized cell-surface expression quantification of 6 ORs as S5 Fig. Normalized cell-surface expression quantification of 6 ORs co-transfected with or without different combinations of RTP members. Transfection with the vector pCI was used as a control divided by all of the read-out values. The grey dotted line represents an arbitrary minimum threshold value for determining cell-surface expression. The *y*-axis represents normalized luminescence value shown as mean ± S.E.M. (*N* = 3).
(TIF)

S1 Table A list of compounds used on the screened ORs. (PDF)

S2 Table Quantification of the subcellular localization of ORs co-transfected with different types of RTPs. Four representative ORs from both of the two categories (MOR180-1, MOR203-1, MOR23-1, and MOR256-17) were transfected with or without the accessory proteins RTP1S, RTP2, or the combination of the two in HEK293T cells. For the columns labeled “ER” and “Golgi”, the results shown are the number of cells in each counting session with OR signals colocalized with the markers for ER or Golgi in permeablized immunocytochemistry. For the columns labeled “Cell-surface”, the results shown are the number of cells seen on the cell-surface that also expressed GFP in live-cell immunocytochemistry. The numbers represent mean ± S.E.M. from three independent counting sessions.
(TIF)

S3 Table Quantification of the subcellular localization of RTP1S and RTP2. For the rows labeled “ER” and “Golgi”, the results shown are the number of cells in each counting session with signals for RTP1S/RTP2 colocalized with the markers for ER or Golgi in permeablized immunocytochemistry. For the rows labeled “Cell-surface”, the results shown are the number of cells seen on the cell-surface that also expressed GFP in live-cell immunocytochemistry. The numbers represent mean ± S.E.M. from three independent counting sessions.
(TIF)
