# Supplementary figures and images for: Receptor-transporting protein (RTP) family members play divergent roles in the functional expression of odorant receptors
Source: PLoS One. 2017 Jun 6;12(6):e0179067. doi: 10.1371/journal.pone.0179067 (PMC5460901; doi:10.1371/journal.pone.0179067)

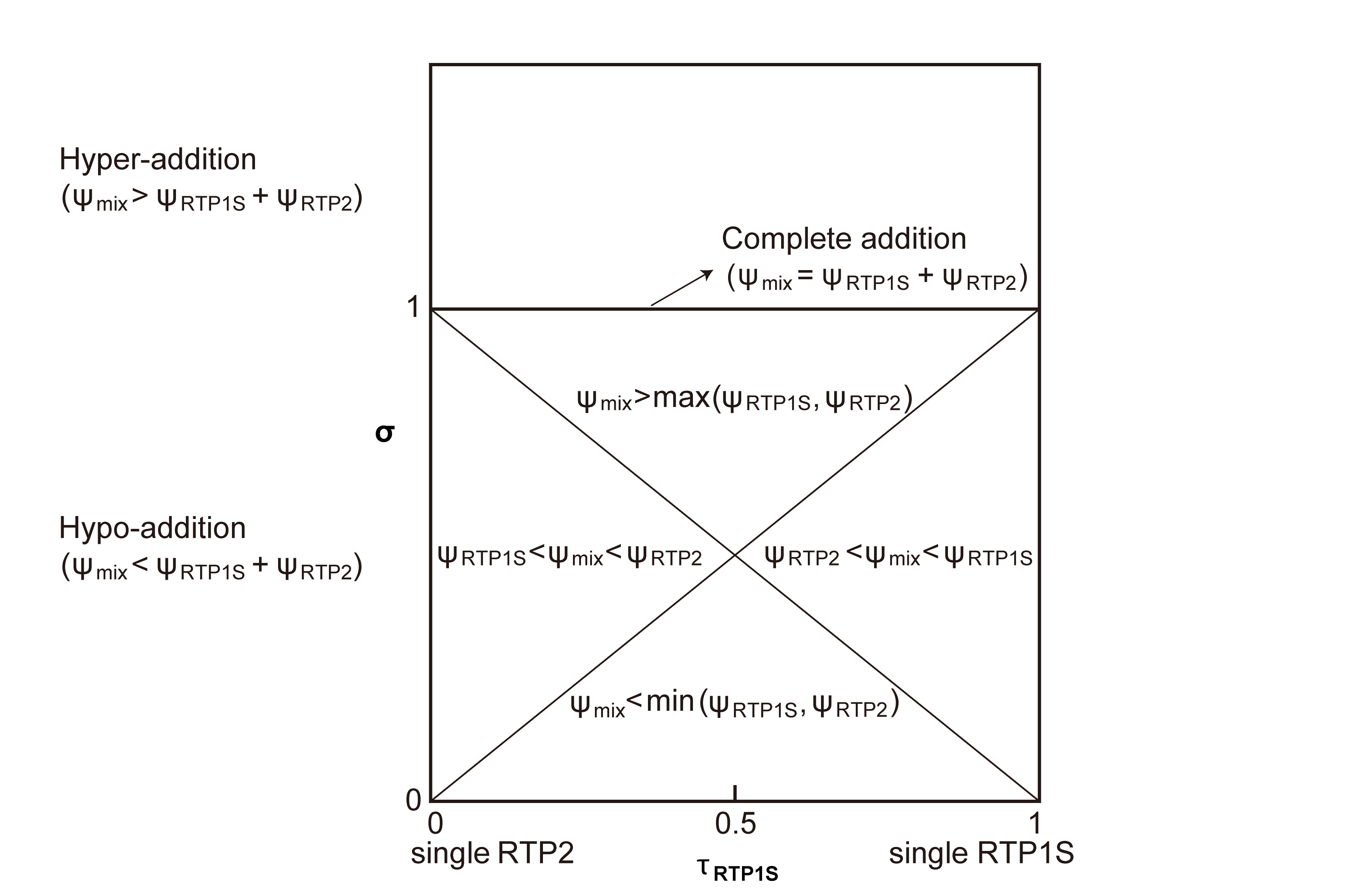

Supplement: S1 Fig — The x-axis represents the value of the parameter τRTP1S, which equals to ψRTP1S / (ψRTP1S + ψRTP2). The y-axis represents the value of the parameter σ, which equals to ψmix / (ψRTP1S + ψRTP2). ψRTP1S, ψRTP2, and ψmix equal to the OR response levels to ligands when co-transfected with RTP1S, RTP2, or the combination of the two, respectively. The combination of RTP1S and RTP2 induced three types of functional interactions, hyper-addition when σ > 1, complete addition when σ = 1, and hypo-addition when σ < 1. The spaces separated by diagonals in the hypo-addition section reflect the relationships among ψRTP1S, ψRTP2, and ψmix. (TIF) [file pone.0179067.s001.tif]

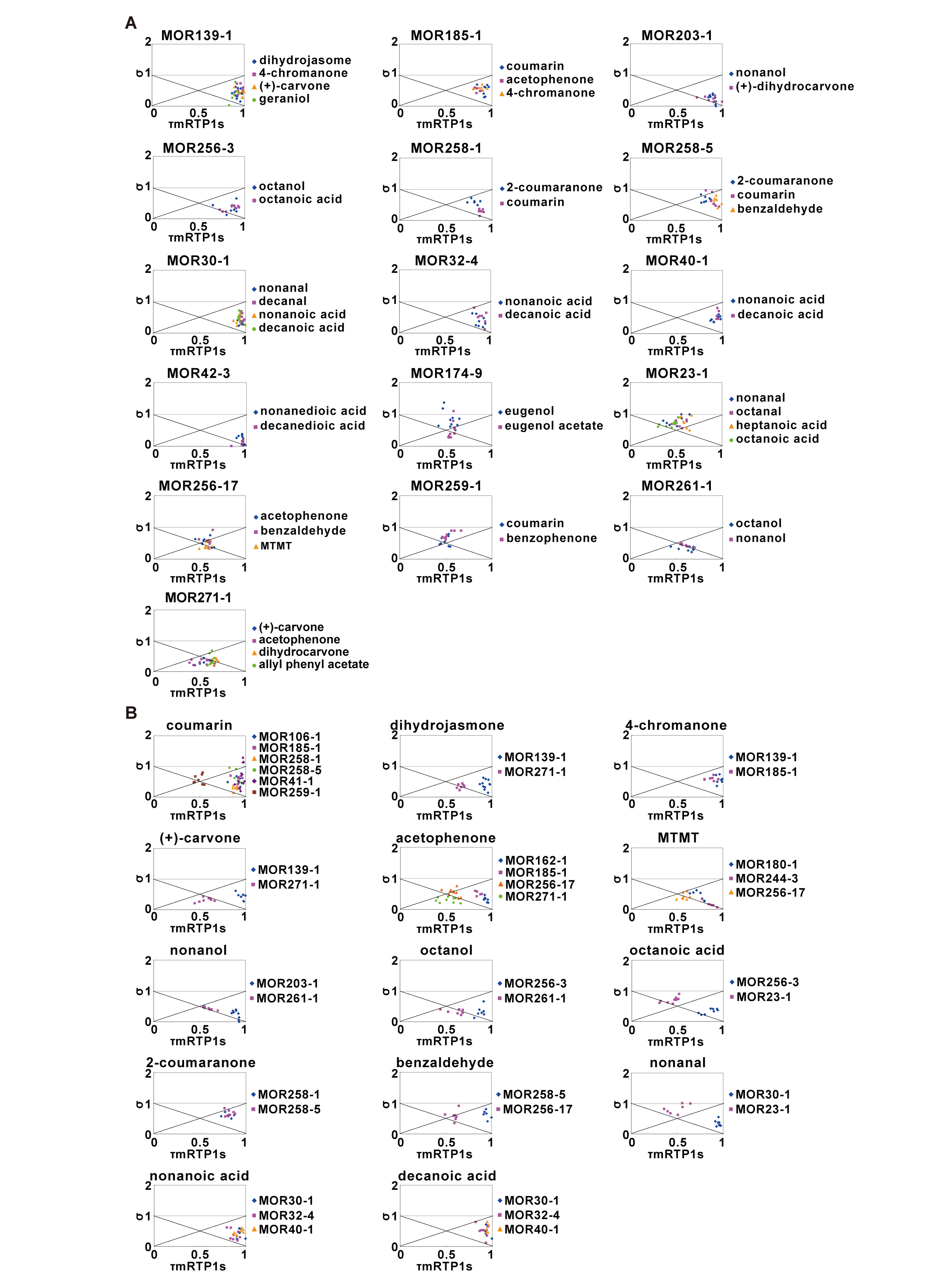

Supplement: S2 Fig — (A) characterization of the functional effect induced by the combination of RTP1S and RTP2 as compared to RTP1S or RTP2 alone for 16 ORs tested against various odorants. The overlaying spatial positions in each of the inset scatter diagrams represent the functional interactions between RTP1S andRTP2 for a certain OR against different odorants. (B) characterization of the functional effect induced by the combination of RTP1S and RTP2 as compared to RTP1S or RTP2 alone for 14 odorants tested against different types of ORs. The overlaying spatial positions in each of the inset scatter diagrams represent the functional interactions between RTP1S and RTP2 for a certain odorant tested against different types of ORs. See Fig 1 for the original dose-response curves. (TIF) [file pone.0179067.s002.tif]

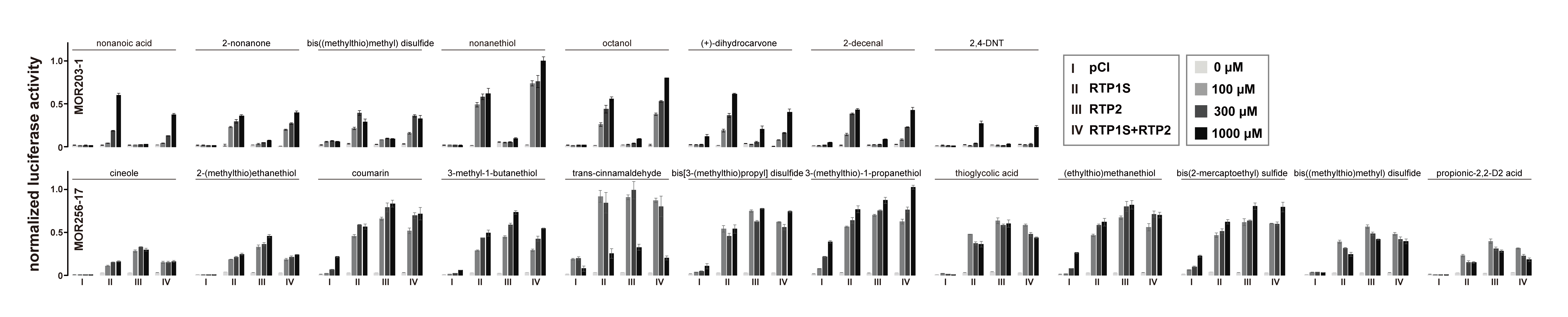

Supplement: S3 Fig — Normalized luciferase activities of representative ORs from Category 1 (MOR203-1) and Category 2 (MOR256-17), transfected alone (I) or co-transfected with different combinations of RTPs, including RTP1S (II), RTP2 (III), and a combination of the two (IV), and tested against the same subsets of ligands as in Fig 2. Different shades of grey columns represent ligand concentrations (0, 100, 300, and 1000 μM). The y-axis represents normalized luciferase activity shown as mean ± S.E.M. (N = 3). (TIF) [file pone.0179067.s003.tif]

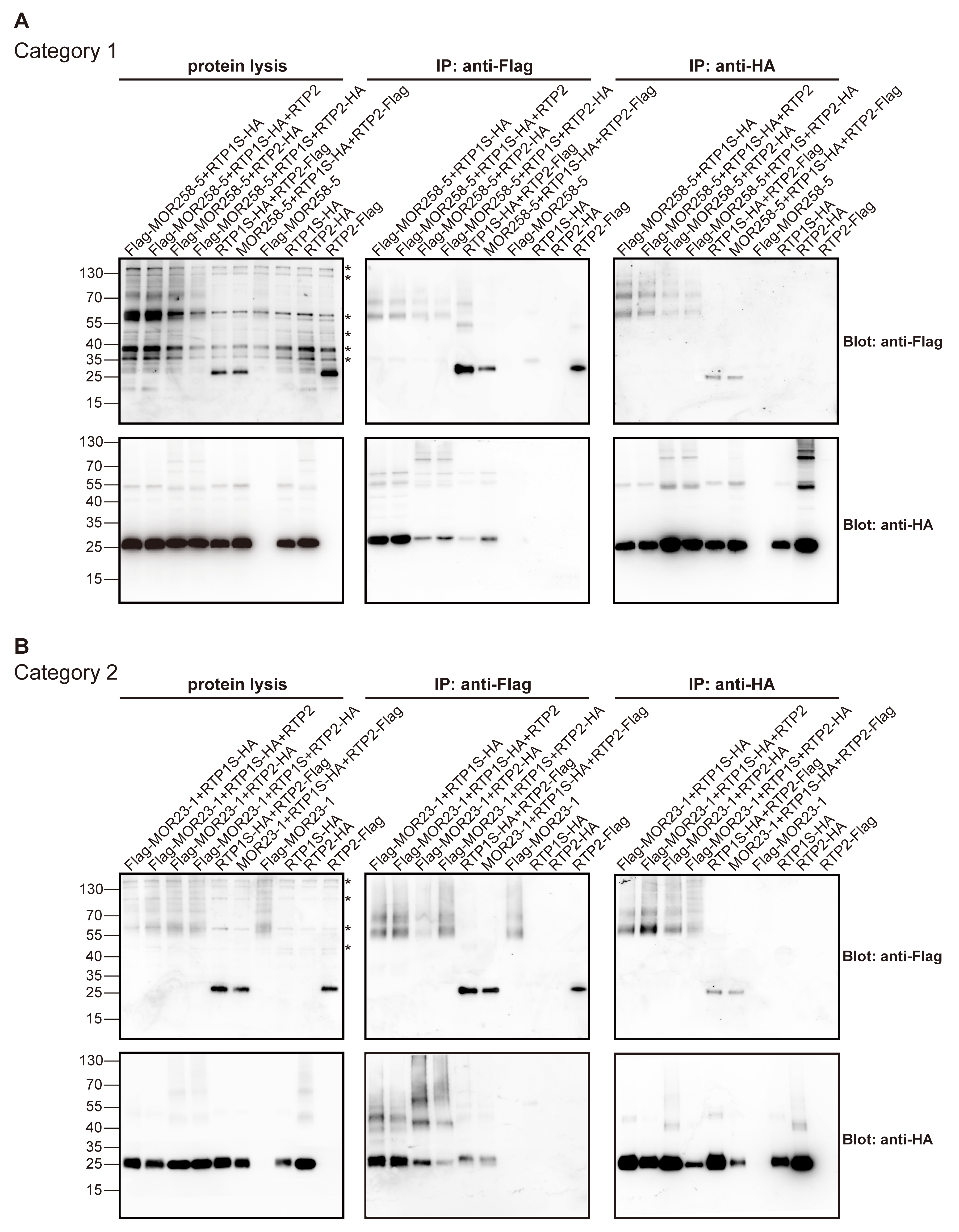

Supplement: S4 Fig — (A) co-immunoprecipitation among MOR258-5 (Category 1), RTP1S, and RTP2. Left panels, protein lysates of HEK293T cells transfected with Flag-tagged MOR258-5 and/or HA-tagged RTP1S and Flag-tagged/HA tagged RTP2; middle panels, co-immunoprecipitation of HA-tagged proteins with anti-Flag antibody; right panels, co-immunoprecipitation of Flag-tagged proteins with anti-HA antibody. (B) co-immunoprecipitation among MOR23-1 (Category 2), RTP1S, and RTP2. Left panels, protein lysates of HEK293T cells transfected with Flag-tagged MOR23-1 and/or HA-tagged RTP1S and Flag-tagged/HA-tagged RTP2; middle panels, co-immunoprecipitation of HA-tagged proteins with anti-Flag antibody; right panels, co-immunoprecipitation of Flag-tagged proteins with anti-HA antibody. The asterisks indicate non-specific bands. (TIF) [file pone.0179067.s004.tif]

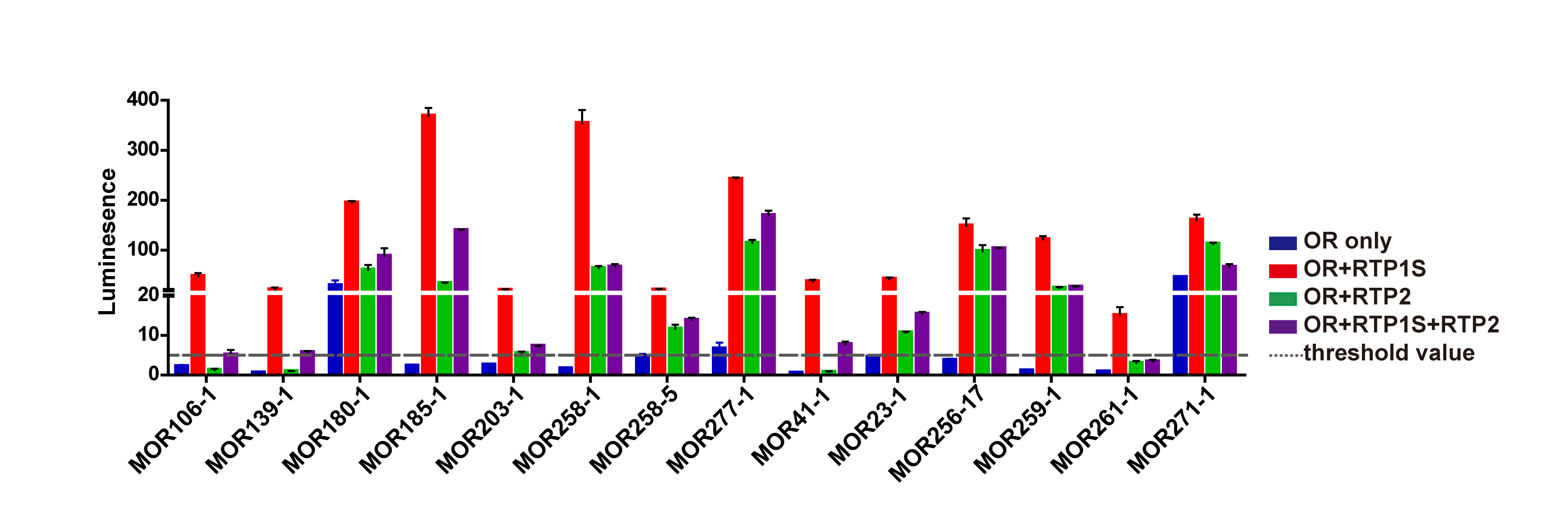

Supplement: S5 Fig — Normalized cell-surface expression quantification of 14 ORs cotransfected with or without different combinations of RTP members including RTP1S (red), RTP2 (green), and a combination of the two (purple). An “OR only” negative control is cotransfected with the empty pCI vector (blue). Transfection with the vector pCI was used as a control divided by all of the read-out values. The grey dotted line represents an arbitrary minimum threshold value for determining cell-surface expression. The y-axis represents normalized luminescence value shown as mean ± S.E.M. (N = 3). (TIF) [file pone.0179067.s005.tif]

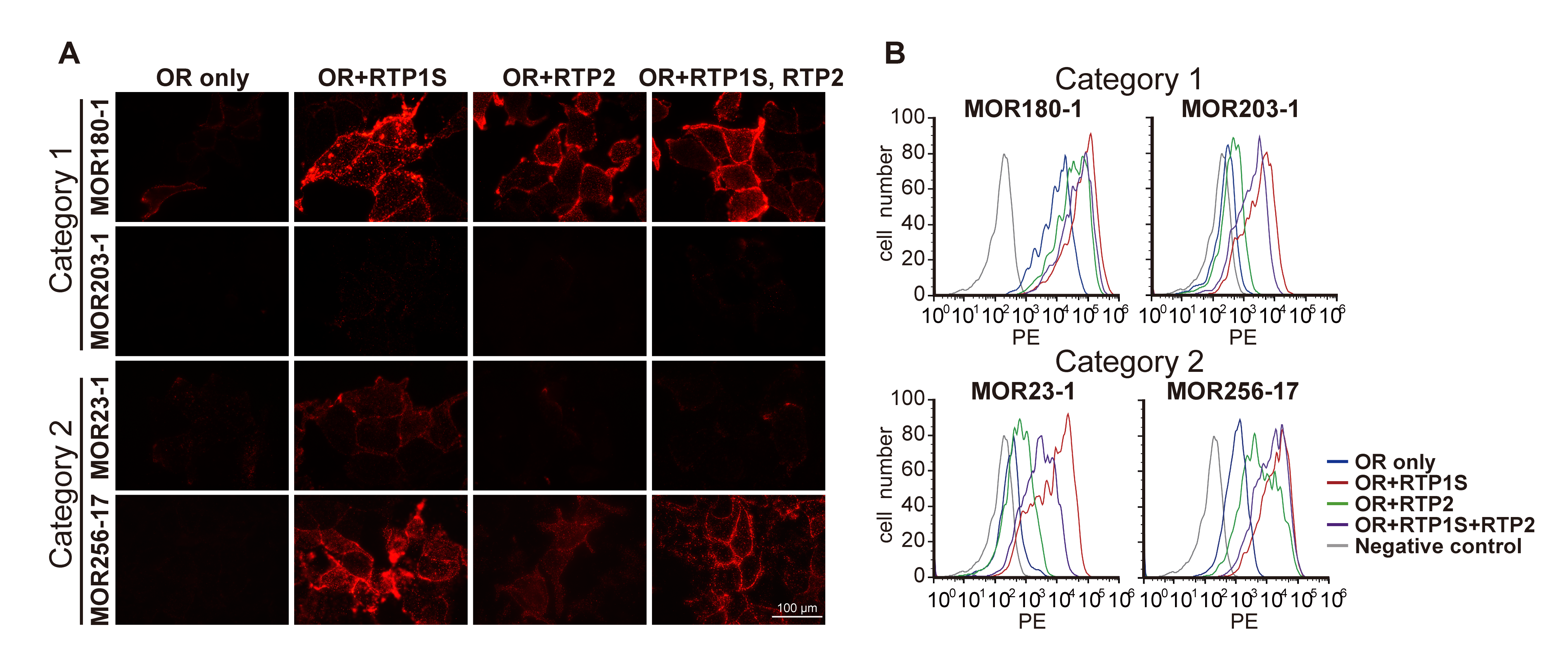

Supplement: S6 Fig — (A) 4 representative ORs from both of the two categories (MOR180-1, MOR203-1, MOR23-1 and MOR256-17) were transfected with or without the accessory proteins RTP1S, RTP2, or the combination of the two in HEK293T cells. Cell-surface fluorescent OR signals are seen as punctate staining. Scale bar, 100 μm. (B) flow cytometry analysis of the cell-surface expression of the 4 ORs with or without the accessory proteins. Transfection with the empty pCI vector was used as a negative control. The intensity of phycoerythrin (PE) signal among the GFP-positive population was measured and plotted. (TIF) [file pone.0179067.s006.tif]

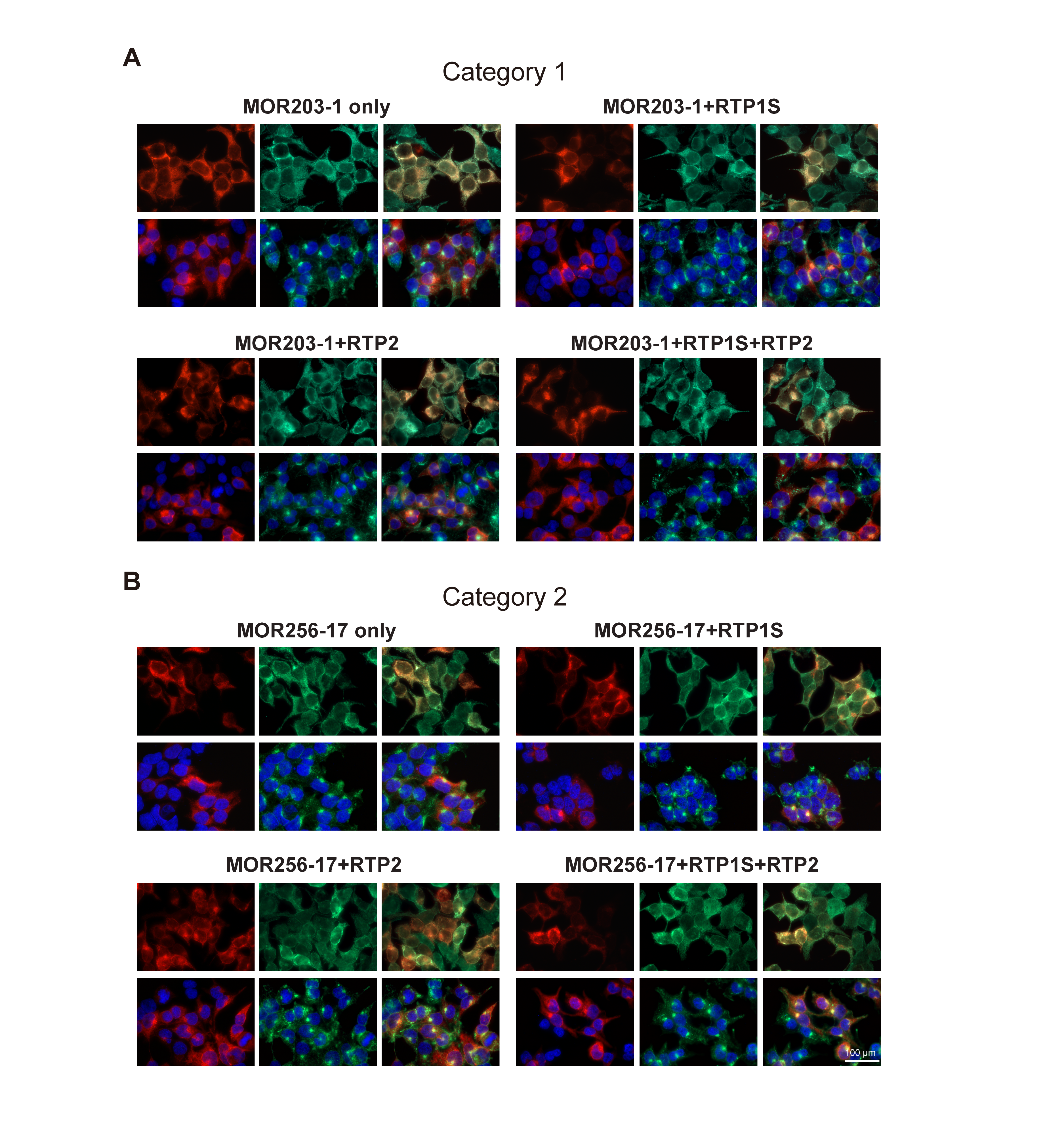

Supplement: S7 Fig — (A-B) subcellular localization of MOR203-1 (Category 1) and MOR256-17 (Category 2) when they were transfected alone or co-transfected with different RTPs or a combination of the two RTPs. Left panels, red signals represent the localization of OR proteins. Middle panels, green signals represent staining of organelles; first row, staining of ER; second row, staining of Golgi. Right panels, yellow signals represent OR proteins merged with the corresponding organelle. Blue signals in the second row are DAPI nuclear staining. Scale bar, 100 μm. (TIF) [file pone.0179067.s007.tif]

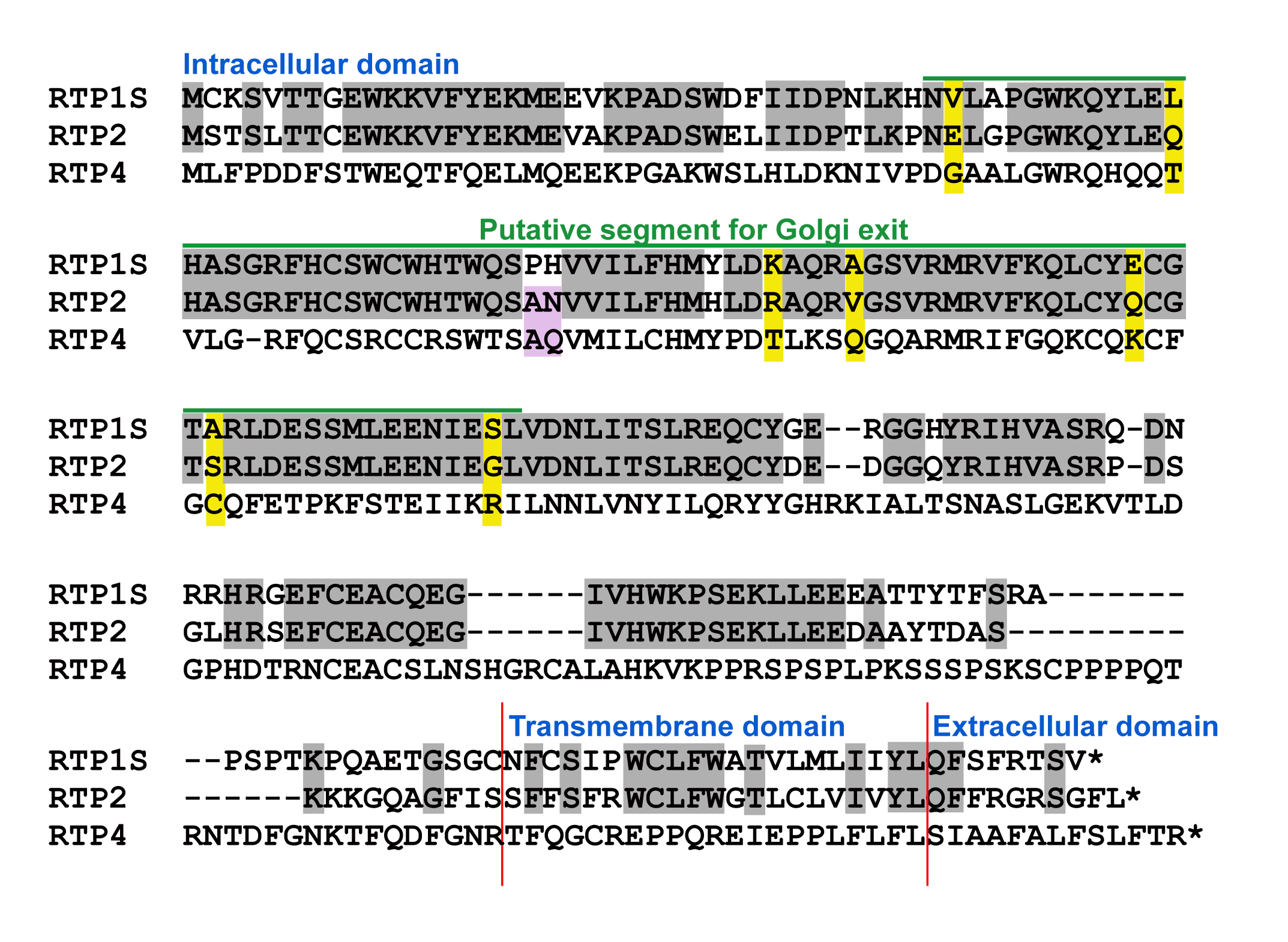

Supplement: S8 Fig — Intracellular, transmembrane, and extracellular domains are delimited with red vertical lines. The residues shaded in grey are conserved between RTP1S and RTP2 (74% identity). The green line spans residues 38 to 117 of RTP1S, representing the middle segment putative for Golgi exit. In this segment, residues shaded in purple are conserved or similar in the side chain properties between RTP2 and RTP4, and residues shaded in yellow are different among RTP1S, RTP2, and RTP4. (TIF) [file pone.0179067.s008.tif]

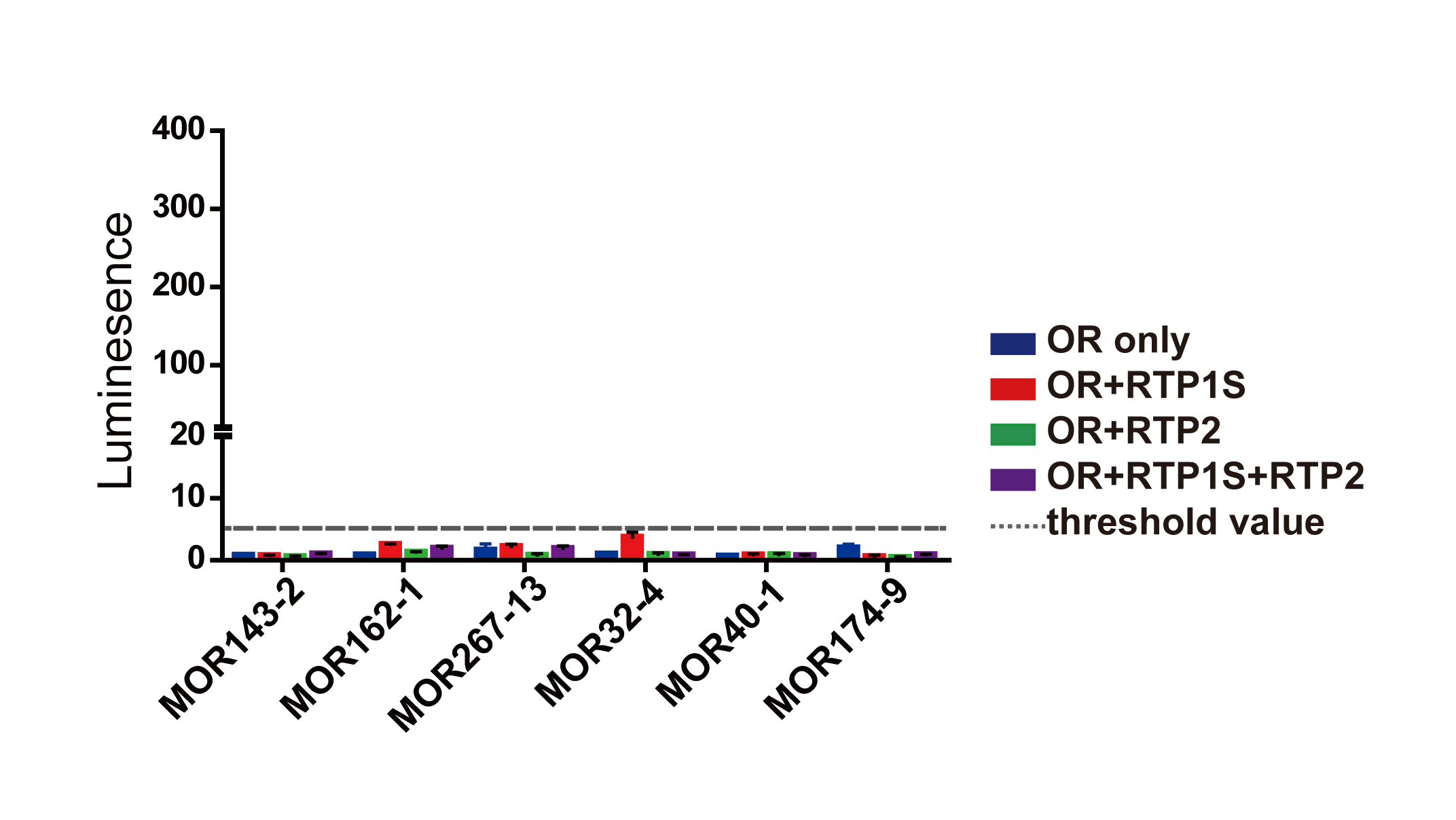

Supplement: S9 Fig — Normalized cell-surface expression quantification of 6 ORs co-transfected with or without different combinations of RTP members. Transfection with the vector pCI was used as a control divided by all of the read-out values. The grey dotted line represents an arbitrary minimum threshold value for determining cell-surface expression. The y-axis represents normalized luminescence value shown as mean ± S.E.M. (N = 3). (TIF) [file pone.0179067.s009.tif]

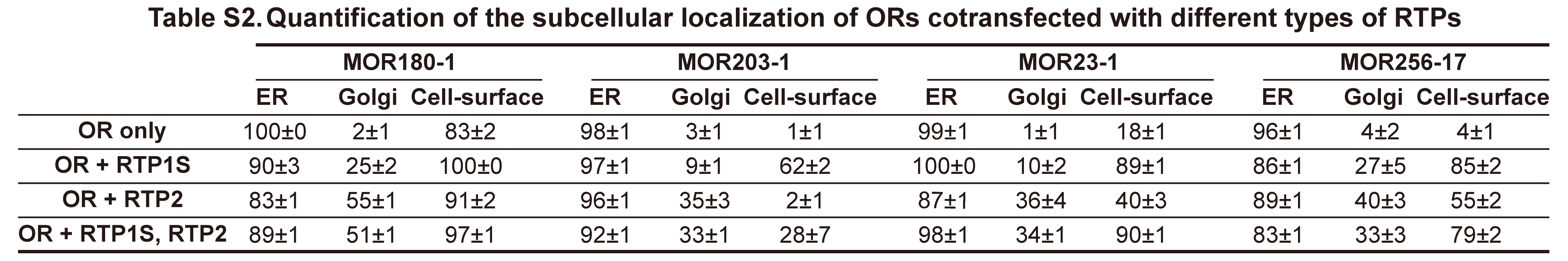

Supplement: S2 Table — Four representative ORs from both of the two categories (MOR180-1, MOR203-1, MOR23-1, and MOR256-17) were transfected with or without the accessory proteins RTP1S, RTP2, or the combination of the two in HEK293T cells. For the columns labeled “ER” and “Golgi”, the results shown are the number of cells in each counting session with OR signals colocalized with the markers for ER or Golgi in permeablized immunocytochemistry. For the columns labeled “Cell-surface”, the results shown are the number of cells seen on the cell-surface that also expressed GFP in live-cell immunocytochemistry. The numbers represent mean ± S.E.M. from three independent counting sessions. (TIF) [file pone.0179067.s011.tif]

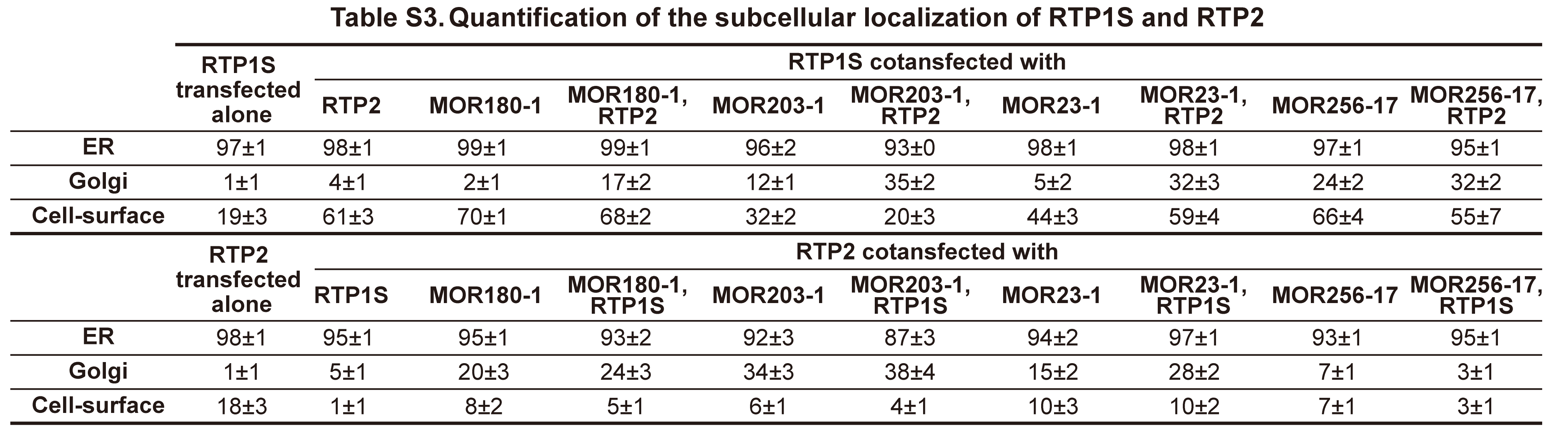

Supplement: S3 Table — For the rows labeled “ER” and “Golgi”, the results shown are the number of cells in each counting session with signals for RTP1S/RTP2 colocalized with the markers for ER or Golgi in permeablized immunocytochemistry. For the rows labeled “Cell-surface”, the results shown are the number of cells seen on the cell-surface that also expressed GFP in live-cell immunocytochemistry. The numbers represent mean ± S.E.M. from three independent counting sessions. (TIF) [file pone.0179067.s012.tif]
