# Supplementary material for: Receptor-transporting protein (RTP) family members play divergent roles in the functional expression of odorant receptors
Source: PLoS One. 2017 Jun 6;12(6):e0179067. doi: 10.1371/journal.pone.0179067 (PMC5460901; doi:10.1371/journal.pone.0179067)
Supplement: S1 Table — (PDF) [file pone.0179067.s010.pdf]

Table S1. A list of compounds used on the screened ORs

common name	IUPAC name	CAS#	source
coumarin	chromen-2-one	91-64-5	Sigma-Aldrich
dihydrojasmone	3-methyl-2-pentylcyclopent-2-en-1-one	1128-08-1	SAFC
4-chromanone	2,3-dihydrochromen-4-one	491-37-2	Adamas
(+)-carvone	(5S)-2-methyl-5-prop-1-en-2-ylcyclohex-2-en-1-one	2244-16-8	Sigma-Aldrich
geraniol	(2E)-3,7-dimethylocta-2,6-dien-1-ol	106-24-1	Sigma-Aldrich
androstenone	5α-androst-16-en-3-one	18339-16-7	Sigma-Aldrich
acetophenone	1-phenylethanone	98-86-2	Sigma-Aldrich
MTMT	methylsulfanylmethanethiol	29414-47-9	synthesis
nonanol	nonan-1-ol	143-08-8	Adamas
(+)-dihydrocarvone	2-methyl-5-prop-1-en-2-ylcyclohexan-1-one	7764-50-3	Fluka
octanol	octan-1-ol	111-87-5	Sigma-Aldrich
octanoic acid	octanoic acid	124-07-2	Sigma-Aldrich
2-coumaranone	3H-1-benzofuran-2-one	553-86-6	Sigma-Aldrich
benzaldehyde	benzaldehyde	100-52-7	Adamas
lyral	4-(4-hydroxy-4-methylpentyl)cyclohex-3-ene-1-carbaldehyde	31906-04-4	Sigma-Aldrich
(-)-camphor	(1S,4S)-4,7,7-trimethylbicyclo[2.2.1]heptan-3-one	464-48-2	Sigma-Aldrich
nonanal	nonanal	124-19-6	Sigma-Aldrich
decanal	decanal	112-31-2	Sigma-Aldrich
nonanoic acid	nonanoic acid	112-05-0	Sigma-Aldrich
decanoic acid	decanoic acid	334-48-5	Sigma-Aldrich
nonanedioic acid	nonanedioic acid	123-99-9	Sigma-Aldrich
decanedioic acid	decanedioic acid	111-20-6	Alfa Aesar
eugenol	2-methoxy-4-prop-2-enylphenol	97-53-0	Sigma-Aldrich
eugenol acetate	(2-methoxy-4-prop-2-enylphenyl) acetate	93-28-7	Sigma-Aldrich
octanal	octanal	124-13-0	Sigma-Aldrich
heptanoic acid	heptanoic acid	111-14-8	Sigma-Aldrich
benzophenone	diphenylmethanone	119-61-9	Sigma-Aldrich
allyl phenyl acetate	prop-2-enyl 2-phenylacetate	1797-74-6	Sigma-Aldrich
galaxolide	4,6,6,7,8,8-hexamethyl-1,3,4,7-tetrahydrocyclopenta[g]isochromene	1222-05-5	Sigma-Aldrich
2-methyl-3-furanthiol	2-methylfuran-3-thiol	28588-74-1	SAFC
bipyridine disulfide	2-[(pyridin-2-ylmethyldisulfanyl)methyl]pyridine	2127-04-0	synthesis
(ethylthio)methanethiol	ethylsulfanylmethanethiol	29414-49-1	synthesis
(methylthio)methanethiol	1-methylsulfanylethanethiol	31331-53-0	synthesis
bis((methylthio)methyl) disulfide	methylsulfanyl-(methylsulfanylmethyldisulfanyl)methane	85544-38-3	synthesis
cinnamaldehyde	(E)-3-phenylprop-2-enal	104-55-2	Sigma-Aldrich
cineole	2,2,4-trimethyl-3-oxabicyclo[2.2.2]octane	470-82-6	Sigma-Aldrich
eugenol methyl ether	1,2-dimethoxy-4-prop-2-enylbenzene	93-15-2	Sigma-Aldrich
dially trisulfide	3-(prop-2-enyltrisulfanyl)prop-1-ene	2050-87-5	Adamas
(methylsulfanyl)methane	disulfide, methyl (methylthio)methyl	42474-44-2	synthesis
1,3-bis(methylseleno)propane	1,3-bis(methylselanyl)propane	6136-91-0	synthesis
2-mercaptopyridine	1H-pyridine-2-thione	2637-34-5	Sigma-Aldrich
2-nonanone	nonan-2-one	821-55-6	Sigma-Aldrich
2-decenal	dec-2-enal	3913-71-1	Alfa Chemistry
nonanethiol	nonane-1-thiol	1455-21-6	Sigma-Aldrich
2,4-DNT	1-methyl-2,4-dinitrobenzene	121-14-2	Sigma-Aldrich
guaiacol	2-methoxyphenol	90-05-1	Sigma-Aldrich
hexanal	hexanal	66-25-1	SAFC
prenylacetate	3-methylbut-2-enyl acetate	1191-16-8	SAFC
butyl acetate	butyl acetate	123-86-4	Sigma-Aldrich
terpineol	2-(4-methylcyclohex-3-en-1-yl)propan-2-ol	98-55-5	Sigma-Aldrich
2-(methylthio)ethanethiol	2-(methylthio)ethanethiol	22322-43-6	synthesis
propionic-2,2-D2 acid	2,2-dideuteriopropanoic acid	19136-91-5	Sigma-Aldrich
bis(2-mercaptoethyl) sulfide	2-(2-sulfanylethylsulfanyl)ethanethiol	3570-55-6	synthesis
3-methyl-1-butanethiol	3-methylbutane-1-thiol	541-31-1	Sigma-Aldrich
thioglycolic acid	2-sulfanylacetic acid	68-11-1	Sigma-Aldrich
3-(methylthio)-1-propanethiol	3-methylsulfanylpropane-1-thiol	26718-09-2	synthesis
bis[(methylsulfinyl)methyl] disulfide	bis[(methylsulfinyl)methyl] disulfide	244171-21-9	synthesis
bis[3-(methylthio)propyl] disulfide	bis[3-(methylthio)propyl] disulfide	85055-65-8	synthesis
